# Efficacy of a new biosynthetic bacterial 6-phytase on growth performance, bone mineralization, and nutrient digestibility in nursery and growing pigs

**DOI:** 10.3389/fvets.2025.1591214

**Published:** 2025-08-22

**Authors:** Maamer Jlali, Clémentine Hincelin, Celsa Manceaux, Sarper Ozbek

**Affiliations:** ^1^Adisseo France S.A.S., European Laboratory of Innovation, Science and Expertise, Saint-Fons, France; ^2^Adisseo France S.A.S., Antony, France; ^3^Parc Technologique du Zoopôle, Zootests, Ploufragan, France

**Keywords:** efficacy, phytase, mineralization, piglets, pigs

## Abstract

Two studies were carried out to investigate the effects of a novel bacterial biosynthetic 6-phytase on growth performance, bone mineralization, and apparent total digestibility (ATTD) of phosphorus (P) in weaned piglets and growing pigs. They were carried out on 192 weaned piglets with initial body weight (BW): 9.3 ± 1.2 kg (33 days of age) and 360 growing pigs with initial BW: 33.3 ± 4.9 kg (85 days) for 43 and 84 days, respectively, according to a randomized complete block design with three treatments. The treatments were a positive control (PC) diet formulated to meet nutrient requirements, a negative control (NC) diet reduced similarly in calcium (Ca) and digestible P by 0.12% points in piglets and by 0.14, 0.11, and 0.10% points, respectively, in phases 1, 2, and 3 in growing pigs, compared to the PC diet; and the NC diet supplemented with the new 6–phytase at 500 phytase units (FTU) per kg of diet (PHY). The mineral depletion decreased final BW (−6.3%, *p* = 0.005; −3.0%, *p* < 0.05), average daily gain (ADG: −8.8%, *p* = 0.003; −4.3%, *p* < 0.05), bone ash content (−24.7%, *p*<*0.001;* −9.6%, *p* = 0.005), bone P content (−24.6%, *p* < 0.001; −6.5%, *p* = 0.11), and ATTD of P (−11.8% points, *p* < 0.001; −9.2% points, *p* < 0.001) and increased the feed–to–gain (F:G) ratio (+2.1%, *p* < 0.001; +4.7%, *p* < 0.05) in weaned piglets and growing pigs, respectively. Compared to animals fed the NC diet, phytase addition improved the final BW (+6.5%, *p* = 0.006, +2.3%, *p* < 0.05), ADG (+9.0%, *p* = 0.005, +3.4%, *p* < 0.05), F:G ratio (−3.1%, *p* < 0.001, −2.4%, *p* < 0.05), metacarpal ash content (+27.6%, *p* < 0.001, +9.3%, *p* = 0.004), and metacarpal P content (+29.3%, *p* < 0.001, +7.0%, *p* = 0.06) in weaned piglets and growing pigs, respectively. The final BW, ADG, and bone ash content in animals fed the NC diet supplemented with phytase were comparable to animals fed the PC diet. The supplementation of phytase to the NC diet improved (P < 0.001) the ATTD of P by 13.5 and 24.6% points (*p* < 0.001) in weaned piglets and growing pigs, respectively. Compared to the NC diet, phytase supplementation also improved the ATTD of Ca (+7.8% points, p) and N (+2.5% points) in growing pigs. This finding indicates the ability of this novel biosynthetic bacterial 6–phytase to restore performance and bone mineralization in piglets and growing pigs fed P– and Ca–reduced diets.

## 1 Introduction

Phosphorus (P) present in plant-feed ingredients constitutes the major part (50% to 75%) of the phosphorus supply in swine diets. However, a significant proportion of the total P (up to 80%) is present in the form of phytate-P, which is poorly digested by non-ruminant animals due to the low level of endogenous phytase activity present in the gastrointestinal tract (GIT) of these animals (1–3).

It is well known that phytate can form complexes with protein and minerals within the GIT, reducing the digestibility and absorption of nutrients in monogastric animals (4, 5). Exogenous phytase is well-known to hydrolyze the phytate complex present in most oilseeds, protein meals, and cereals (6, 7). Hence, supplementation of swine diets with microbial phytase has largely proven to be an effective way to enhance the bioavailability of P from phytate-P, improving P digestibility, performance, and bone mineralization of animals fed diets reduced in P and calcium (Ca) and reducing the excretion of phosphorus in the environment (8–10). However, the effects of such exogenous phytase on these parameters could largely depend on its source, dose, and intrinsic characteristics of the evaluated phytase and feed composition, especially in terms of dietary P and Ca levels. A novel bacterial 6-phytase characterized by a high affinity for myo-inositol hexakisphosphate (IP6) and other myo-inositol phosphate (IP) esters, a high activity over a wide pH range, and a high intrinsic thermostability, which is important during feed processing, was recently developed. In addition, its efficacy in broilers and laying hens was also investigated (10, 11). However, there are limited data to demonstrate the efficacy of this phytase in nursery and growing pigs. Therefore, the aim of this study was to investigate the effects of this novel bacterial 6-phytase on growth performance, bone mineralization, and apparent total tract digestibility (ATTD) of nutrients in nursery and growing pigs fed diets reduced in digestible P and Ca.

## 2 Materials and methods

All experimental procedures were carried out according to legislation governing the ethical treatment of animals, and investigators were certified by the French government to conduct animal experiments. All experimental procedures were conducted under the authorization number G-03-159-4 at the Center of Expertise in Research and Nutrition (Adisseo France SAS, Malicorne, France) and internal standard operating procedures at Zootests (Ploufragan, France).

### 2.1 Animals, diets, and experimental design

#### 2.1.1 Experiment 1—Weaned piglets

The experiment was conducted at the Center of Expertise in Research and Nutrition (Adisseo France SAS, 03600 Malicorne). A total of 192 weanling male piglets (initial BW: 9.3 ±1.2 kg) were blocked by initial body weight and randomly allotted to three dietary treatments using a randomized complete block design. Dietary treatments consisted of a positive control (PC) diet with adequate supply of all nutrients according to the National Research Council (NRC) 2012 including Ca (0.70 and 0.66% for phases 1 and 2, respectively) and digestible P (0.33 and 0.31% for phases 1 for 14 days and 2 for 28 days, respectively); a negative control (NC) reduced in Ca and digestible P similarly by 0.12% unit (as the amount of digestible P which can be released with the present phytase at 500 FTU/kg diet) compared to the PC diet; and a NC diet supplemented with this new 6-phytase at 500 FTU/kg diet (Rovabio PhyPlus, Adisseo France SAS, France). The composition and nutrient characteristics of the basal diets are presented in Table 1. Titanium dioxide was included in all diets of phase 2 (post-weaning 2) at 5 g/kg as an indigestible marker for apparent total tract digestibility (ATTD) of P assessment. Diets were offered as pellets throughout the experimental period. Piglets had *ad libitum* access to experimental diets and water for 42 d. The body weight (BW) and feed intake (FI) were recorded two times (at days 14 and 42) to determine the average daily gain (ADG), average daily feed intake (ADFI), and feed-to-gain (F:G) ratio. Digestibility measurements were carried out on days 34 to 38 of the experiment, and fresh fecal samples were collected every day from 16 pens per treatment, then pooled by pen, and immediately stored at −20°C until further analysis and determination of the total tract digestibility (ATTD) of P. On day 43, all piglets (24 piglets per treatment) from 12 pens per treatment used for fecal sample collection were anesthetized by intramuscular injection of Zoletil 100 (1 mL/10 kg BW) and then euthanized by T61 (1 mL/10 kg BW), and the right feet from the fore were excised to determine metacarpal bones (III and IV) ash and P contents.

**Table 1:** Composition and characteristics of basal diets for weanling piglets (Experiment 1).

	**Phase 1 (post-weaning 1)**		**Phase 2 (post-weaning 2)**	
	**PC**	**NC**	**PC**	**NC**
**Composition, %**				
Corn	45.63	45.45	58.95	60.31
Wheat	12.28	13.79	15.00	15.00
Soybean meal 48%	27.39	27.09	19.27	19.04
Whey powder	10.00	10.00	-	-
Rapeseed oil	1.00	0.60	1.91	1.47
Monocalcium phosphate	0.79	0.12	1.19	0.52
Calcium carbonate	0.55	0.53	0.68	0.66
L-Lysine HCl 98%	0.43	0.44	0.42	0.42
DL-methionine 99%	0.13	0.12	0.07	0.06
L-Threonine 98.5%	0.13	0.13	0.12	0.12
L-Tryptophan 98%			0.02	0.02
Salt	0.24	0.24	0.43	0.43
Premix^a^	1.45	1.45	1.45	1.45
Titanium dioxide	-	-	0.50	0.50
**Calculated nutrients, %**				
Dry matter	88.51	88.38	87.52	87.37
Crude protein	19.09	19.12	15.75	15.75
Crude fat	3.36	2.97	4.55	4.16
Crude fiber	2.96	2.97	2.85	2.87
Ash	5.39	4.84	4.62	4.07
NE, kcal/kg	2,412	2,412	2,475	2,475
Dig. Lysine	1.23	1.23	0.98	0.98
Dig. Methionine	0.39	0.39	0.29	0.29
Dig. Cystine	0.29	0.29	0.26	0.26
Dig. Met+Cys	0.68	0.68	0.55	0.55
Dig. Threonine	0.73	0.73	0.59	0.59
Dig. Tryptophan	0.20	0.20	0.17	0.17
Dig. Arginine	1.10	1.09	0.88	0.88
Dig. Valine	0.78	0.78	0.64	0.64
Calcium	0.70	0.58	0.66	0.54
Total P	0.60	0.45	0.59	0.44
Digestible P	0.33	0.21	0.31	0.19
Phytate-P	0.21	0.22	0.21	0.22
Sodium	0.17	0.17	0.17	0.17

^a^Premix provides per kg feed: vitamin A, 9715 IU; vitamin D3, 1943 IU; vitamin E (α-tocopherol), 48.4 IU; vitamin B1, 1.9 mg; vitamin B2, 4.8 mg; vitamin B3, 19.5 mg; vitamin B5, 14.5 mg; vitamin B6, 2.9 mg; vitamin B12, 29 μg; vitamin K3, 1.9 mg; Fe, 135 mg; I, 0.73 mg; Cu, 87 mg; Mn, 58.4 mg; Zn, 103 mg; Se, 0.30 mg; choline, 290 mg. PC, positive control diet; NC, negative control diet similarly reduced by 0.12% unit of digestible P and Ca.

#### 2.1.2 Experiment 2—Growing pigs

The experiment was conducted at Zootests SAS (Ploufragan, France). A total of 360 growing pigs (180 barrows and 180 gilts) with an average initial BW of 33.4 ± 4.9 kg (85 days of age) and individually identified with radio-frequency identification (RFID) tags were divided into two runs and assigned to three treatments in a randomized complete block design. Dietary treatments consisted of a positive control (PC) diet with adequate supply of all nutrients including Ca and digestible P; a negative control (NC) reduced in digestible P (0.17 vs. 0.31, 0.15 vs. 0.26, and 0.13 vs. 0.23% for phases 1, 2, and 3, respectively) and Ca (0.55 vs. 0.69, 0.50 vs. 0.61, and 0.46 vs. 0.56% for phases 1, 2, and 3, respectively) compared to the PC diet; and a NC diet supplemented with the new 6-phytase at 500 FTU/kg diet (Rovabio PhyPlus, Adisseo France SAS, France). Experimental diets were fed in three phases (grower 1 for 29 days, grower 2 for 25 days, and finisher for 28 days). The composition and characteristics of basal diets are presented in Table 2. Each treatment was presented equally in each block (*n* = 4 blocks with 15 pigs/treatment in each block). Each block was equipped with two Self Automatic Feeders (SAF) that allow for recording daily individual body weight and individual feed consumption. Titanium dioxide was included in all diets of phase 1 at 5 g/kg as an indigestible marker for ATTD of nutrients. From days 109 to 113 (phase 1) of the experiment, fresh fecal samples were collected by rectal stimulation from 16 pigs per treatment during 5 consecutive days, then pooled by pig, and immediately stored at −20°C until further analysis. At the end of phase 1 (on day 113 of the experiment), 16 pigs were randomly chosen per treatment. They were anesthetized by intramuscular injection of Zoletil 100 (1 mL/10 kg BW) and then euthanized by T61 (1 mL/10 kg BW), and the right feet from the fore were excised to determine metacarpal bones (III and IV) ash and P contents.

**Table 2:** Composition and characteristics of basal diets for growing-finishing pigs (Experiment 2).

	**Phase 1 (from 85 to 115 d)**		**Phase 2 (from 115 to 140 d)**		**Phase 3 (from 140 to 182 d)**	
	**PC**	**NC**	**PC**	**NC**	**PC**	**NC**
**Composition, %**						
Corn	73.74	75.32	81.49	82.674	87.42	86.75
Wheat bran	1.00	1.00	2.00	2.00	4.47	5.92
Soybean meal 48%	19.75	19.48	12.80	12.64	5.19	5.00
Soy oil	1.51	1.00	0.59	0.19	0.00	0.00
Calcium carbonate	0.86	0.84	0.81	0.79	0.79	0.78
Monocalcium phosphate	1.20	0.41	0.95	0.34	0.81	0.24
L-Lysine HCl 98%	0.41	0.42	0.38	0.38	0.37	0.37
DL-Methionine 99%	0.07	0.07	0.03	0.03	0.00	0.00
L-Threonine 98.5%	0.11	0.11	0.10	0.10	0.10	0.10
L-Tryptophan 98%	0.03	0.03	0.03	0.03	0.04	0.04
Salt	0.43	0.43	0.43	0.43	0.41	0.41
Premix^a^	0.40	0.40	0.40	0.40	0.40	0.40
Titanium dioxide	0.50	0.50	-	-	-	-
**Nutrient characteristics, %**						
Dry matter	87.33	87.16	87.04	86.91	86.82	86.76
Crude protein	15.52	15.52	12.95	12.97	10.24	10.32
Crude fat	4.49	4.03	3.76	3.40	3.35	3.37
Crude fiber	2.94	2.96	2.78	2.80	2.69	2.79
Ash	4.65	4.00	4.11	3.60	3.67	3.24
Net energy, kcal/kg	2,475	2,475	2,475	2,475	2,475	2,475
Dig. Lysine	0.98	0.98	0.79	0.79	0.61	0.61
Dig. Methionine	0.30	0.30	0.23	0.23	0.17	0.17
Dig. Met+Cys	0.55	0.55	0.45	0.45	0.36	0.36
Dig. Threonine	0.59	0.59	0.49	0.49	0.40	0.40
Dig. Tryptophan	0.17	0.17	0.14	0.14	0.11	0.11
Dig. Arginine	0.88	0.88	0.69	0.69	0.49	0.49
Dig. Valine	0.64	0.64	0.53	0.53	0.42	0.42
Calcium	0.69	0.55	0.61	0.50	0.56	0.46
Total P	0.59	0.42	0.52	0.38	0.48	0.36
Digestible P	0.31	0.17	0.26	0.15	0.23	0.13
Phytate-P	0.22	0.22	0.22	0.22	0.22	0.23
Sodium	0.17	0.17	0.17	0.17	0.16	0.16

^a^Premix provides per Kg feed: vitamin A, 2,680 IU; vitamin D3, 536 IU; vitamin E (alfa-tocopherol), 13.3 IU; vitamin B1, 0.5 mg; vitamin B2, 1.3 mg; vitamin B3, 5.4 mg; vitamin B5, 4.0 mg; vitamin B6, 0.8 mg; vitamin B12, 8 μg; vitamin K3, 0.5 mg; Fe, 37.3 mg; I, 0.2 mg; Cu,24 mg; Mn, 16.1 mg; Zn, 28.4 mg; Se, 0.1 mg; choline, 80 mg. PC, positive control diet; NC, negative control diet similarly reduced by 0.14, 0.11, and 0.10% units in digestible P and Ca for grower 1, grower 2, and finisher phase, respectively, compared to the PC diet.

### 2.2 Chemical analyses

Representative samples of all experimental diets were analyzed for dry matter, crude fat, ash, crude fiber, nitrogen (N), Ca, total P, phytate-P, and phytase activity. In addition, in Experiment 1, total P was analyzed in fecal samples, and in Experiment 2, total P, Ca, and N were analyzed in fecal samples. Dry matter, crude fat, ash, crude fiber, and N were determined using the method AOAC procedure (method 934.01; AOAC, 2006). Total P and Ca were analyzed using the inductively coupled plasma (ICP) spectroscopy method (method 985.01 A, B, and C, AOAC, 2006). Phytate-P was analyzed using the colorimetric method. The concentration of titanium dioxide in diets and ileal digesta was analyzed following the procedure of Myers et al. (12). Ash content in metacarpal bones was determined by removing the adhering tissue, drying the bone at 110°C for 12 h, and extracting fat with ether. The dry fat-free bones were ashed in a muffle furnace at 550°C for 3 h, according to AOAC International (2000, method 932.A6). P content in metacarpal bones was measured using dry-ashed bone samples. The activity of phytase in the experimental diets was analyzed according to the ISO standard methodology (ISO Standard 300242, 2009). One unit of phytase is defined as the amount of enzyme that liberates one micromole of inorganic orthophosphate from phytic acid per minute at pH 5.5 and 37°C.

### 2.3 Calculations and statistical analysis

The ATTD (%) of P, Ca, and N in the experimental diets was determined using the following equation:


$$\begin{array}{c} \text{ATTD}=\left(1-\left[\left({\text{Ti}}_{\text{d}}/{\text{Ti}}_{\text{f}}\right)\text{ x }\left({\text{N}}_{\text{f}}/{\text{N}}_{\text{d}}\right)\right]\right)\text{ x }100 \end{array}$$

where Ti_d_ and Ti_f_ are the concentration of titanium in diets and feces, respectively; N_d_ and N_f_ are the concentration of nutrients (P, Ca, or N) in diets and feces, respectively.

Data were analyzed using the MIXED procedure of SAS (SAS Inst. Inc., Cary, NC). Residuals were checked for normality. In Experiment 1, block (*n* =32) and treatment (*n* = 3) were used as fixed variables. In Experiment 2, an individual pig was used as an experimental unit. Treatment, gender, and animals' batch, and their interactions were considered as fixed variables. Initial body weights of pigs were used as covariates for growth performance parameters (BW, ADG, ADFI, and FCR). Comparisons of least square means for each significant effect were performed using the Tukey-Kramer test. Statistical significance was set at a *P* < 0.05, and 0.05 < *P* < 0.10 was considered a trend.

## 3 Results

The analyzed values of dietary nutrients and phytase activities in experimental diets are presented in Tables 3, 4, respectively. The analyzed dietary Ca and total P contents were, in general, in agreement with the expected values. Analyzed values of phytate-P were, on average, 0.21% and 0.22% in experiments 1 and 2, respectively. Analyzed phytase activities in the experimental diets supplemented with phytase were 415 (phase 1), 507 (phase 2), and 474 (phase 1), 511 (phase 2), and 501 (phase 3) FTU/kg diet in experiments 1 and 2, respectively. In the two experiments, no mortality was observed in the groups, and all the animals were in good health and readily consumed diets throughout the duration of both experiments.

**Table 3:** Analyzed nutrients of experimental diets (Experiment 1).

**Item**	**Post-weaning 1**			**Post-weaning 2**		
	**PC**	**NC**	**PHY**	**PC**	**NC**	**PHY**
Dry matter, %	90.50	90.20	90.00	87.80	87.50	88.20
Crude protein, %	18.80	19.20	19.00	15.10	15.20	15.20
Crude fat, %	3.00	2.30	2.50	4.10	4.00	3.60
Crude fiber, %	2.30	2.30	2.40	2.00	2.00	2.20
Ash, %	5.10	4.80	4.70	4.20	4.30	4.60
Calcium, %	0.72	0.58	0.61	0.66	0.55	0.54
Total phosphorus, %	0.63	0.47	0.46	0.57	0.43	0.45
Phytate-P, %	0.25	0.22	0.23	0.21	0.20	0.15
Phytase activity, FTU/kg diet	47	82	415	115	112	507

PC, positive control diet; NC, negative control diet; similarly reduced by 0.12% unit of digestible P and Ca; PHY, negative control diet supplemented with phytase (Rovabio PhyPlus) at 500 FTU/kg diet.

**Table 4:** Analyzed nutrients of experimental diets (Experiment 2).

	**Batch 1**									**Batch 2**								
**Item**	**Phase 1**			**Phase 2**			**Phase 3**			**Phase 1**			**Phase 2**			**Phase 3**		
	**PC**	**NC**	**PHY**	**PC**	**NC**	**PHY**	**PC**	**NC**	**PHY**	**PC**	**NC**	**PHY**	**PC**	**NC**	**PHY**	**PC**	**NC**	**PHY**
Dry matter, %	88.70	88.20	87.50	88.00	87.00	87.40	87.40	86.90	86.90	87.60	87.40	87.10	87.40	87.00	86.90	88.60	88.00	88.20
Crude protein, %	14.90	14.90	14.80	12.40	12.40	12.50	10.60	10.60	10.60	15.30	15.10	15.50	12.80	13.30	12.50	10.30	10.50	10.50
Crude fat, %	3.90	4.10	3.70	3.30	2.50	2.60	2.90	2.50	2.50	3.80	3.40	3.10	3.30	2.70	2.90	3.30	3.50	3.30
Crude fiber, %	2.60	2.70	2.90	2.20	2.20	2.30	2.00	2.30	2.10	2.50	3.00	2.80	2.80	2.60	2.80	2.30	2.50	2.50
Ash, %	4.70	4.20	4.20	3.50	3.00	3.00	3.20	2.70	2.70	4.60	4.20	4.10	3.50	3.30	3.10	3.10	2.90	2.80
Calcium, %	0.68	0.49	0.51	0.51	0.48	0.46	0.47	0.43	0.42	0.66	0.54	0.49	0.54	0.48	0.42	0.54	0.38	0.39
Total P, %	0.58	0.40	0.39	0.48	0.36	0.37	0.46	0.35	0.36	0.57	0.39	0.41	0.49	0.38	0.38	0.47	0.34	0.35
Phytate-P, %	0.23	0.21	0.22	0.21	0.20	0.23	0.18	0.22	0.22	0.22	0.25	0.25	0.22	0.22	0.23	0.22	0.22	0.23
Phytase activity, FTU/kg diet	0	118	474	88	82	511	133	93	501	29	23	485	38	64	424	67	63	424

PC, positive control diet; NC, negative control diet, similarly reduced by 0.14, 0.11, and 0.10% units in digestible P and Ca for grower 1, grower 2, and finisher phase, respectively, compared with PC diet; PHY, negative control diet supplemented with phytase (Rovabio PhyPlus) at 500 FTU/kg diet.

### 3.1 Experiment 1—Weaned piglets

The effects of dietary treatments on growth performance, bone mineralization, and ATTD of P in piglets are presented in Tables 5, 6. Piglets fed with the NC diet reduced in digestible P and Ca had lower final body weight (-6.3%, *p* = 0.005), ADG (-8.8%, *p* = 0.003), and ADFI (-6.9%, *p* = 0.02) and a higher F:G ratio (+2.1%, *p* = 0.02) in comparison with piglets fed with the PC diet. In addition, mineral reduction decreased (*p* < 0.001) the ash and P contents in metacarpal bones by 24.7 and 24.6%, respectively. Compared to piglets fed with the NC diet, piglets fed with the NC diet supplemented with phytase at 500 FTU/kg diet had a greater final body weight (+6.5%, *p* = 0.006) and ADG (+9.0%, *p* = 0.005) and a lower F:G ratio (-3.1%, *p* < 0.001). ADFI tended (*p* = 0.06) to be higher in piglets that received phytase-supplemented NC diet than those fed with the NC diet. In addition, phytase supplementation on top of the NC diet improved (*p* < 0.001) bone ash and P contents by 27.6% and 29.3%, respectively, in comparison with the NC diet. There was no significant difference between piglets fed with the NC diet supplemented with phytase and those fed with the PC on final BW (*p* = 0.91), ADG (*p* = 0.83), ADFI (*p* = 0.55), F:G ratio (*p* = 0.18), bone ash content (*p* = 0.19), and bone P content (*p* = 0.13). Piglets fed with the NC diet had lower (*p* < 0.001) ATTD of P as compared to piglets fed with the PC diet. However, the supplementation of phytase to the NC diet improved (*p* < 0.001) the ATTD of P by 13.5% points.

**Table 5:** Growth performance of weaned piglets fed diets without or with phytase for 42 days (Experiment 1).

**Item**	**Treatment**			**Adjusted SEM**	***P*-value**
	**PC**	**NC**	**PHY**		
**Phase 1, 0–14d**					
BW d0, kg	9.25	9.34	9.32	0.03	0.13
BW d14, kg	13.66	13.50	13.99	0.17	0.13
ADG d0-14, g/piglet/d	315.23	297.15	333.92	11.59	0.09
ADFI d0-14, g/piglet/d	515.64	509.05	525.40	11.96	0.63
FCR d0-14	1.672^ab^	1.761^a^	1.590^b^	0.034	0.004
**Phase 2, 14-42d**					
BW d42, kg	36.14^a^	33.86^b^	36.05^a^	0.53	0.006
ADG d14-42, g/piglet/d	802.82^a^	727.10^b^	787.73^a^	15.28	0.003
ADFI d14-42, g/piglet/d	1,398.14^a^	1,286.47^b^	1,365.89^ab^	27.98	0.02
FCR d14-42	1.740^ab^	1.768^a^	1.731^b^	0.01	0.04
**Global period, 0-42d**					
ADG d0-42, g/piglet/d	640.29^a^	583.78^b^	636.46^a^	12.58	0.004
ADFI d0-42, g/piglets/d	1,103.97^a^	1,027.33^b^	1,085.73^ab^	21.23	0.04
FCR d0-42	1.724^b^	1.760^a^	1.705^b^	0.01	0.001

^a, b^Within a row, least square means without a common superscript letter differ (p < 0.05).

PC, positive control diet; NC, negative control diet; similarly reduced by 0.12% unit of digestible P and Ca; PHY, negative control diet supplemented with phytase (Rovabio PhyPlus) at 500 FTU/kg diet; SEM, standard error of the mean.

**Table 6:** Bone traits of weaned piglets fed diets without or with phytase for 42 days (Experiment 1).

**Item**	**Treatment**			**Adjusted SEM**	***P*-value**
	**PC**	**NC**	**PHY**		
ATTD of P	58.01^a^	46.19^b^	59.67^a^	0.89	< 0.001
Bone dry matter, %	54.31^a^	51.99^b^	54.20^a^	0.36	< 0.001
Bone ash, g	5.13^a^	3.87^b^	4.93^a^	0.11	< 0.001
Bone ash, %	18.39^a^	14.96^c^	17.44^b^	0.21	< 0.001
Bone ash, % DM	33.86^a^	28.78^c^	31.92^b^	0.30	< 0.001
Bone P, g	0.896^a^	0.676^b^	0.874^a^	0.02	< 0.001
Bone P, %	3.21^a^	2.62^b^	3.09^a^	0.04	< 0.001
Bone P, % DM	5.84^a^	5.03^b^	5.66^a^	0.07	< 0.001

^a, b, c^Within a row, least square means without a common superscript letter differ (p < 0.05).

PC, positive control diet; NC, negative control diet; similarly reduced by 0.12% unit of digestible P and Ca; PHY, negative control diet supplemented with phytase (Rovabio PhyPlus) at 500 FTU/kg diet; SEM, standard error of the mean; DM, dry matter.

### 3.2 Experiment 2—Growing pigs

The effects of dietary treatments on growth performance, bone mineralization, and ATTD of nutrients in growing pigs are presented in Tables 7, 8. No significant interaction was observed between dietary treatment and sex on growth performance parameters, bone traits, and ATTD of nutrients. Growing pigs fed with the NC diet reduced in digestible P and Ca had lower (*p* < 0.05) final BW (−3.0%) and ADG (−4.3%) and a higher F:G ratio (+4.7%) in comparison with piglets fed the PC diet. In addition, digestible P and Ca reduction decreased (*p* = 0.005) the ash content in metacarpal bones by 9.6%. Bone P content tended to be lower (−6.5%, *p* = 0.11) in pigs that received the NC diet in comparison with pigs fed with that received the PC diet. Compared to growing pigs fed with the NC diet, pigs fed with the NC diet supplemented with phytase at 500 FTU/kg diet had a greater (*p* < 0.05) final body weight (+2.3%) and ADG (+3.4%) and a lower F:G ratio (−2.4%). In addition, phytase supplementation on top of the NC diet improved (*p* < 0.05) bone ash content by 9.3% and tended (*p* = 0.06) to improve P content by 7.0%, in comparison with the NC diet. There was no significant difference between pigs fed with the NC diet supplemented with phytase and those fed with the PC diet on final BW, ADG, ADFI, bone ash content, and bone P content. However, the F:G ratio remains higher (*p* < 0.05) in pigs fed with the phytase-supplemented diet than those fed the PC diet. Regarding the digestibility of nutrients, digestible P and Ca reduction decreased (*p* < 0.001) the ATTD of P by 9.2% points, increased (*p* = 0.01) ATTD of Ca by 8.3% points, and maintained the ATTD of N. However, the supplementation of phytase improved (*p* < 0.05) the ATTD of P (+24.6% points), Ca (+7.8% points), and N (+2.5% points) in comparison with the NC diet.

**Table 7:** Growth performance of growing pigs fed diets without or with phytase for 84 days (Experiment 2).

	**Treatment**			**Adjusted**	***P*** **-value**					
**Item**	**PC**	**NC**	**PHY**	**SEM**	**Initial BW**	**Treatment (T)**	**Sex (S)**	**Batch (B)**	**T x S**	**T x B**
**Phase 1**										
BW 85d, kg	33.3	33.4	33.3	0.260	-	0.99	0.53	0.003	0.98	1.00
BW 114d, kg	61.4^a^	59.2^b^	60.5^a^	0.496	< 0.001	< 0.001	< 0.001	< 0.001	0.32	0.76
ADG 85-114d, kg/pig/d	0.962^a^	0.888^b^	0.931^a^	0.010	< 0.001	< 0.001	< 0.001	< 0.001	0.32	0.76
ADFI 85-114d, kg/pig/d	2.14	2.09	2.14	0.021	< 0.001	0.408	< 0.001	0.05	0.08	0.43
FCR 85-114d	2.25^b^	2.37^a^	2.32^a^	0.018	< 0.001	0.002	0.21	< 0.001	0.31	0.34
**Phase 2**										
BW 140d, kg	86.6^a^	84.0^b^	85.3^ab^	0.590	< 0.001	0.004	< 0.001	0.18	0.92	0.90
ADG 115-140d, kg/piglet/d	0.983	0.966	0.974	0.007	< 0.001	0.501	< 0.001	< 0.001	0.75	0.85
ADFI 115-140d, kg/piglet/d	2.74	2.74	2.74	0.022	< 0.001	0.970	< 0.001	< 0.001	0.66	0.41
FCR d115-140	2.79	2.85	2.82	0.015	< 0.001	0.261	0.02	0.13	0.88	0.63
**Phase 3**										
BW d169, kg	109.2^a^	105.9^b^	108.3^a^	0.631	< 0.001	0.002	< 0.001	0.02	0.83	0.83
ADG d141-169, kg/piglet/d	0.784	0.785	0.804	0.007	< 0.001	0.3696	< 0.001	< 0.001	0.73	0.51
ADFI d141-169, kg/piglet/d	2.79^b^	2.86^ab^	2.90^a^	0.021	< 0.001	0.035	< 0.001	< 0.001	0.76	0.05
FCR d141-169	3.60	3.69	3.66	0.025	< 0.001	0.287	0.21	0.13	0.29	0.80
**Global period**										
ADG d85-169, kg/pig/d	0.903^a^	0.864^b^	0.893^a^	0.006	< 0.001	0.002	< 0.001	0.02	0.83	0.83
ADFI d85-169, kg/pig/d	2.472	2.471	2.498	0.017	< 0.001	0.620	< 0.001	< 0.001	0.87	0.10
FCR d85-169	2.74^c^	2.87^a^	2.80^b^	0.012	< 0.001	< 0.001	0.09	< 0.001	0.59	0.10

^a, b, c^Within a row, least square means without a common superscript letter differ (p < 0.05).

PC, positive control diet; NC, negative control diet; similarly reduced by 0.12% unit of digestible P and Ca; PHY, negative control diet supplemented with phytase (Rovabio PhyPlus) at 500 FTU/kg diet; SEM, standard error of the mean.

**Table 8:** Bone traits and apparent total digestibility of phosphorus, calcium, and nitrogen in growing pigs fed diets without or with phytase for 84 days (Experiment 2).

	**Treatment**			**Adjusted**	***P*** **-value**				
**Item**	**PC**	**NC**	**PHY**	**SEM**	**Treatment (T)**	**Sex (S)**	**Batch (B)**	**T x S**	**T x B**
**Bone traits**									
Dry matter, %	62.5	61.1	62.2	0.29	0.10	0.82	0.04	0.57	0.50
Bone ash, g	8.3^a^	7.5^b^	8.2^a^	0.11	0.00	0.42	< 0.001	0.48	0.94
Bone ash, % DM	35.2^a^	33.7^b^	35.2^a^	0.31	0.02	0.05	< 0.001	0.42	0.95
Bone P, g	1.38	1.29	1.38	0.02	0.07	0.33	0.11	0.68	0.73
Bone P, % DM	5.90	5.70	5.90	0.06	0.43	0.45	0.53	0.40	0.63
**ATTD**									
Phosphorus	43.63^b^	34.42^c^	59.06^a^	1.76	< 0.001	0.12	0.87	0.65	0.26
Calcium	43.49^c^	51.62^b^	59.41^a^	1.41	< 0.001	0.10	0.43	0.62	0.13
Nitrogen	74.26^b^	75.84^b^	78.37^a^	0.57	< 0.001	< 0.001	< 0.001	0.37	0.93

^a, b, c^Within a row, least square means without a common superscript letter differ (p < 0.05).

PC, positive control diet; NC, negative control diet; similarly reduced by 0.12% unit of digestible P and Ca; PHY, negative control diet supplemented with phytase (Rovabio PhyPlus) at 500 FTU/kg diet; SEM, standard error of the mean.

## 4 Discussion

The first phytases were commercialized in 1990, and within a decade, phytases were being added routinely to swine diets to break down phytic acid, thereby increasing the availability of P, improving growth performance, and reducing the excretion of P into the environment (13). However, the benefits of adding exogenous phytases to livestock feeds, especially in pigs, can mainly depend on their source and dose as well as on the nutritional characteristics of diets. Growth performance is often used as an indicator of phytase efficacy on P utilization, particularly when animals are fed P-deficient diets (9, 14). As expected, the dietary reduction in digestible P and Ca reduced growth performance in piglets and growing pigs in comparison with animals fed a P- and Ca-adequate diet. These results are in agreement with many previous studies (9, 15–17). Similarly, Lee et al. (18) reported that the growth performance of pigs was reduced when dietary Ca and P were below the requirement. These results indicate the essentiality of P and Ca to optimize the productivity of piglets and growing pigs. Indeed, P is the second most abundant bodily mineral next to calcium (19). It is an essential nutrient required for normal muscle growth, and it plays vital roles in maintaining osmotic and acid–base balance, energy metabolism, and protein synthesis (19, 20). It appears also from these results that reducing P and Ca levels by 0.12% points from the NRC (2012) recommended values in piglets and growing pigs has a negative impact on growth performance. Otherwise, several studies have reported improvements in performance when phytase was used in piglets and growing pigs (21–24).

In the current study, the addition of phytase at 500 FTU/kg diet to a diet similarly reduced in digestible P and Ca by 0.12% units fed to weaned piglets improved the final BW, ADG, and F:G ratio by 6.5%, 9.0%, and 3.1%, respectively. These results indicate the ability of phytase to release P and Ca and probably the other nutrients, which therefore improves the growth performance of nursery and growing pigs. In the current study, the improvements in growth performance parameters observed with phytase supplementation were lower in growing pigs compared to those in weaned piglets. Indeed, final BW, ADG, and FCR were improved by 2.3%, 3.4%, and 2.4%, respectively, in growing pigs fed a phytase-supplemented diet in comparison with those fed an NC diet. These differences in terms of improvements may be because weaned piglets are more sensitive to P and Ca reduction compared to growing pigs, and they need these minerals for skeletal development and mineralization. As previously reported by Cambra-Lopez et al. (25) and recently by Babatunde and Adeola (9), the age of pigs can influence the efficacy of phytase on growth performance. It is important to notice in the current study that the magnitude of the efficacy of phytase on growth performance seems to be reduced during the growing period. Several other factors, such as diet composition, dietary phytate-P level, and source and dose of phytase can also influence the magnitude of phytase response in nursery and growing pigs (21, 22, 26). It is also important to highlight that in the experiment performed on growing pigs, phytase supplementation at 500 FTU/kg diet was able to partially restore the F:G ratio compared to pigs fed the nutrient adequate-PC diet. This observation could be explained by the negative effect of a high reduction in digestible P and Ca, especially during the first grower phase (similarly by 0.14% points) on the F:G ratio, which was translated during the whole experimental period. The bone ash and P content results in piglets and growing pigs are consistent with the growth performance results. Many studies have demonstrated that dietary Ca and P play an indispensable role in the bone development and therefore on bone ash content of piglets and pigs (20, 27, 28). As observed on growth performance, there was a negative effect of dietary reduction in P and Ca on ash content in metacarpal bones of piglets and growing pigs. This could be an indicator that reducing digestible P and Ca similarly by 0.12% points from NRC (2012) recommended level in weaned piglets and growing pigs was sufficient to induce negative effects not only on growth performance but also on bone mineralization. In agreement with previous studies (23, 29, 30), phytase supplementation at 500 FTU/kg diet in P- and Ca-deficient diets improved the metacarpal bones ash and P contents in weaned piglets and growing pigs. The levels of metacarpal bones ash and P contents in animals fed with the phytase-supplemented diets became equivalent to those fed with the PC-nutrient adequate diets. This observation indicates the effectiveness of this new biosynthetic 6-phytase to improve the utilization of P and Ca in pigs fed the P- and Ca-deficient diets. Indeed, growing pigs fed with the low P and Ca diet had higher ATTD of Ca than those offered adequate P and Ca diet. A higher Ca utilization at a lower dietary Ca concentration has already been shown by Varley et al. (31) and Dersjant-Li and Dusel (32) in growing pigs fed diets reduced in Ca and digestible P. It seems that the absorption and use efficiency of dietary Ca increases when less Ca is provided. In the present study, the result that 6-phytase supplementation increased the ATTD of P in Experiment 1 and ATTD of P and Ca in Experiment 2 is expected and it agrees with the results from previous experiments (9, 33–36). These improvements of P and Ca digestibility could be explained by the mechanism of action of phytase on phytate which is probably efficient to release or remove minerals, especially Ca, from the phytate bond as well as limiting the interactions between nutrients and phytate. However, Bournazel et al. (37) reported that addition of a microbial phytase at 500 FTU/kg diet did not influence the ATTD of Ca but increased the retainable Ca in growing pigs. These different outcomes may be due to phytase type and dose, Ca level and source, as well as the Ca:P ratio which all these factors may influence the response of phytase on ATTD of Ca. In addition to P and Ca, phytase addition also improved the ATTD of N. These results confirm that phytate may chelate not only dietary Ca but also protein and basic amino acids such as lysine, arginine, and histidine, but when phytate is hydrolyzed by exogenous phytase, the chelated protein and amino acids will be released and absorbed, which will increase the utilization of proteins next to minerals (38). As reported by several studies (21, 23, 39), the phytases from the new generation (from bacterial origin) are highly efficient at low pH especially at the upper part of gastrointestinal tract (GIT) which may lead to limit the nutrient–phytate complexes within the GIT and improving thereby the bioavailability of nutrients including minerals and amino acids for monogastric animals. However, some limitations of these two studies including the efficacy of this novel phytase depending on its dose and nutritional conditions (different energy and nutrient density) can be highlighted. Therefore, further studies are needed to elucidate the mode of action of this new biosynthetic 6-phytase according to dietary phytate level and its dose on the bioavailability of nutrients beyond P, Ca, and N in monogastric animals.

## 5 Conclusion

In conclusion, this study confirmed that reducing digestible P and Ca similarly by 0.12% points in weaned piglets and by 0.14, 0.11, and 0.10% points, in phases 1, 2, and 3, respectively, in growing pigs, from the NRC (2012) recommended level, was sufficient to induce negative effects on growth performance, bone mineralization, and P digestibility in both weaned piglets and growing pigs. The addition of the novel biosynthetic 6-phytase to P- and Ca-deficient diets fed to weaned piglets and growing pigs has the potential to improve nutrient digestibility and, therefore, enhance performance and bone mineralization.

## Data Availability

The raw data supporting the conclusions of this article will be made available by the authors, without undue reservation.
